# Alternating Diet as a Preventive and Therapeutic Intervention for High Fat Diet-induced Metabolic Disorder

**DOI:** 10.1038/srep26325

**Published:** 2016-05-18

**Authors:** Yongjie Ma, Mingming Gao, Dexi Liu

**Affiliations:** 1 Department of Pharmaceutical and Biomedical Sciences, College of Pharmacy, University of Georgia, Athens, Georgia 30602, USA

## Abstract

This study presents the alternating diet as a new strategy in combating obesity and metabolic diseases. Lean or obese mice were fed a high-fat diet (HFD) for five days and switched to a regular diet for one (5 + 1), two (5 + 2), or five (5 + 5) days before switching back to HFD to start the second cycle, for a total of eight weeks (for prevention) or five weeks (for treatment) without limiting animals’ access to food. Our results showed that animals with 5 + 2 and 5 + 5 diet alternations significantly inhibited body weight and fat mass gain compared to animals fed an HFD continuously. The dietary switch changed the pattern of daily caloric intake and suppressed HFD-induced adipose macrophage infiltration and chronic inflammation, resulting in improved insulin sensitivity and alleviated fatty liver. Alternating diet inhibited HFD-induced hepatic *Pparγ*-mediated lipid accumulation and activated the expression of *Pparα* and its target genes. Alternating diet in the 5 + 5 schedule induced weight loss in obese mice and reversed the progression of metabolic disorders, including hepatic steatosis, glucose intolerance, and inflammation. The results provide direct evidence to support that alternating diet represents a new intervention in dealing with the prevalence of diet-induced obesity.

Obesity is a “modern” disease caused by changes in lifestyle and dietary structure, although genetics also plays an important role. Excess food intake (especially foods enriched with fat and sugar nutrients) and a sedentary lifestyle are the major contributors to obesity^1^. Obesity is associated with many diseases, including diabetes, cardiovascular diseases, fatty liver, cancer, and other metabolic complications^2^.

Disequilibrium between energy intake and energy expenditure is the major cause of the obesity epidemic. Therefore, dietary control, by reducing energy intake, has been considered a rational approach to limiting weight gain^3^,^4^. Studies have reported successful short-term effects in controlling weight gain under preclinical or clinical settings, validating these approaches and their underlying principles^5^,^6^,^7^,^8^. However, these successes have been limited to a small percentage of individuals^9^ and fasting has not been proven highly effective long-term due to food restriction induced-hyperphagia, resulting in poor compliance^10^. Therefore, effective dietary interventions that are practical, easy to adapt, and capable of promoting long-term adherence are urgently needed to at first slow down and eventually prevent the worldwide obesity epidemic.

Continuous high fat diet (HFD) feeding causes nutrition imbalance, resulting in multiple metabolic abnormalities. Previous studies have reported that switching mice from obesogenic diets to regular diets, without limiting food intake, was capable of inducing weight loss and improving metabolic syndrome^11^. However, animals regained the weight when they were placed back on HFD and long-term benefits could not be obtained. In this study, we cycled an HFD with a regular diet without control of food consumption and evaluated the effectiveness of an alternating diet strategy (high fat diet followed by regular diet) in managing obesity and metabolic disorders. Using C57BL/6 mice as an animal model, we characterized the growth, glucose and lipid metabolisms, and energy homeostasis of animals kept on an HFD *ad lib* feeding for five days and switched to a regular diet for either one, two, or five days, again *ad lib*, before switching back to an HFD repeatedly, for a total of eight weeks. In addition, we also applied this strategy in a pre-existing obesity model for a total of five weeks to evaluate the therapeutic effect. The results showed that animals given the (5 + 2) or (5 + 5) dietary alternation significantly inhibited diet-induced weight gain and adiposity, and also improved liver steatosis and glucose intolerance. Moreover, the alternating diet also reversed pre-existing obesity and metabolic disturbance, suggesting that an alternating diet without restricting food intake represents a promising strategy in dealing with obesity epidemics.

## Results

### Alternating diet suppresses HFD-induced weight gain

**Figure 1:**
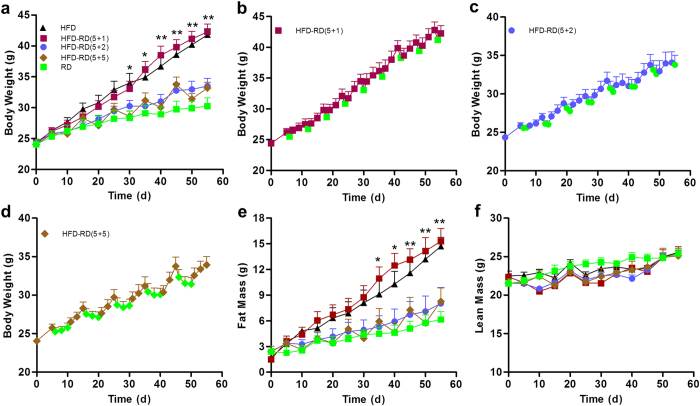
An alternating diet suppresses HFD-induced body weight and fat mass gain. Mice were fed an HFD, a regular diet, or an alternating diet for 8 weeks. (**a**) Body weight of animals on different diets as a function of time. (**b**) Body weight-time curve of mice on alternating diet with 5 days on HFD and 1 day on regular diet (5 + 1). (**c**) Body weight-time curve of mice on alternating diet with 5 days on HFD and 2 days on regular diet (5 + 2). (**d**) Body weight-time curve of animals on alternating diet with 5 days on HFD and 5 days on regular diet (5 + 5). Green dots in panels (**b–d**) represent the body weight of mice obtained when animals were on a regular diet. (**e**) Fat mass and (**f**) lean mass were analyzed once every 5 days using EchoMRI-100^TM^ System. Data represent mean ± SEM (n = 10). *P < 0.05, **P < 0.01 compared to mice with continuous HFD feeding. Abbreviations: high fat diet (HFD), regular diet (RD).

C57BL/6 mice were fed an HFD for five days intermitted by a regular diet for one, two, or five days. The repeating feeding cycles were continued for a total of eight weeks. Control animals were fed either an HFD or a regular diet continuously without a diet switch. After an eight-week feeding period, animals fed a regular diet showed a body weight of 30 g compared to 42 g for animals on an HFD (Fig. 1a), whereas animals fed a regular diet for more than two days after five days of an HFD showed a body weight similar to that of regular diet-fed control animals and 9 grams less than that of obese mice fed an HFD. Animals with (5 + 1) did not show a significant difference in body weight, suggesting that a minimum of two days on a regular diet is necessary to prevent an HFD-induced weight gain. A detailed analysis of weight gain revealed that the body weight of animals when on a regular diet (green dots in Fig. 1b–d) is always lower than that of animals when on an HFD (dots in other colors in Fig. 1b–d), suggesting that animals lost weight when they were switched from an HFD to a regular diet. Results in Fig. 1e,f showed that weight differences among groups of animals are due to fat mass. Lean mass of animals is the same among the different groups. In other words, diet alternation with schedules of (5 + 2) and (5 + 5) prevented animals from growing fat mass.

### Effect of alternating diet on energy intake

**Figure 2:**
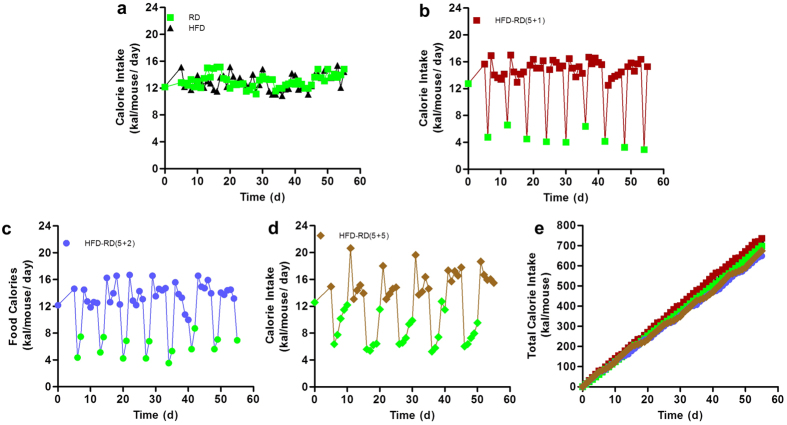
Effects of an alternating diet on daily calorie intake, and accumulating food and energy intake. Mice were fed a regular diet (RD) or high fat diet (HFD) continuously (**a**), or an alternating diet with a schedule of 5 + 1 (**b**), 5 + 2 (**c**) or 5 + 5 (**d**). The green dots in panels (**b–d**) represent caloric intake of mice when on a regular chow. Daily caloric intake was calculated based on daily food intake. Total food (**e**) and calorie intake (**f**) per mouse was calculated based on daily food and caloric intake of HFD (food-energy conversion: HFD, 1 gram = 5.49 kcal; regular diet, 1 gram = 3.46 kcal)

Measurements of daily calorie intake (Fig. 2) showed an initial dramatic drop when switching from an HFD to a regular diet (green dots in Fig. 2b–d), followed by a gradual increase for animals on (5 + 2) and (5 + 5). A significant increase in energy intake was evident when animals were switched back to an HFD (other colored dots in Fig. 2b–d). The patterns of energy intake were consistent in every cycle of dietary alternation, and the fluctuation in energy uptake was similar to the patterns of body weight gain (Fig. 1a). A much smaller fluctuation in caloric intake was seen in animals fed the same diet (Fig. 2a). It is likely that the fluctuation in caloric intake with the alternating diet is responsible, in part, for maintaining body weight. Animals in all of the groups had similar total caloric intake (Fig. 2e). However, if one divides the total energy intake by body weight gained during the feeding period, the total calories per gram of body weight gained by HFD-fed control animals is 40.5 kcal/g, 39.8 kcal/g with (5 + 1), 68.3 kcal/g with (5 + 2), and 73.3 kcal/g with (5 + 5) diet schedule. These data suggest that the feeding efficiency (which equals [average weight gain /average calorie intake] ×100%) in animals on a (5 + 2) and (5 + 5) diet schedule is significantly lower than in the control animals (1.42% and 1.36% vs. 2.45%), thus showing that animals on an alternating diet with a minimum of two days on a regular diet within every five days of an HFD feeding are less efficient in converting the intake energy into body weight or body fat.

### Alternating diet prevents fat accumulation in adipose tissue and inhibits macrophage recruitment in WAT

**Figure 3:**
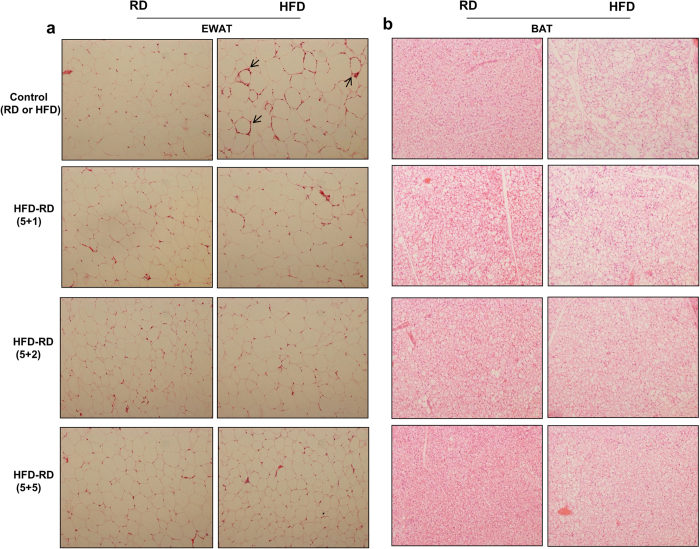
An alternating diet inhibited fat storage in adipocytes of WAT and BAT. Mice were fed an HFD continuously or an alternating diet for 8 weeks. At the end of the experiments, mice with the alternating diet were divided and sacrificed while on either HFD or regular diet feeding. Epididymal white adipose tissue (**a**) and brown adipose tissue (**b**) were collected and H&E staining was performed to examine structure of adipocytes (100×). Arrow: crown-like structure.

By the end of the study, mice on the alternating diet were sacrificed on either the regular diet or at the HFD portion of the cycle. The results in Fig. 3 showed images of the H&E-stained epididymal WAT (EWAT) and BAT. As expected, an HFD significantly induced fat accumulation in adipose tissue, determined by the average size of adipocytes in EWAT and the density of vacuole-type structures in BAT (Fig. 3a,b). In contrast, the average size of adipocytes in animals fed an alternating diet was significantly smaller than that of animals fed an HFD continuously. The difference becomes more evident when mice fed an alternating diet were sacrificed while on a regular diet.

**Figure 4:**
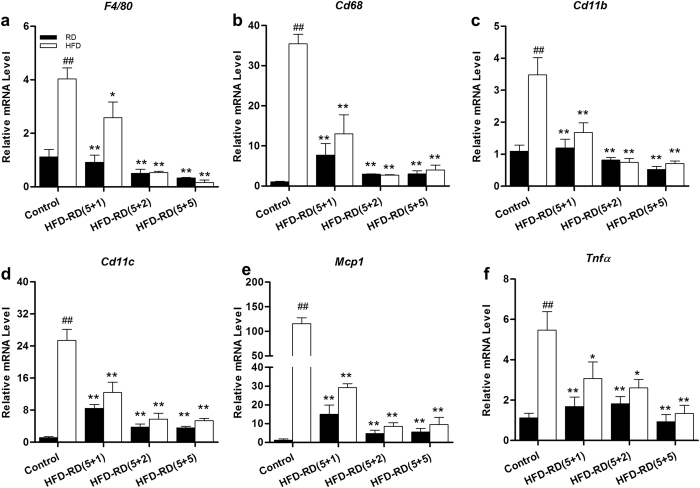
Alternating diet improved diet-induced inflammation in EWAT. Epididymal adipose tissues were harvested after mice were sacrificed when on HFD or regular diet and total RNA was extracted. Relative mRNA levels of macrophage marker genes including *F4/80* (**a**), *Cd68* (**b**), *Cd11b* (**c**), *Cd11c* (**d**), *Tnfα* (**e**) and *Mcp1* (**f**) were determined by real-time PCR. ^##^P < 0.01 compared to mice continuously fed with a regular diet), *P < 0.05, **P < 0.01 compared to mice continuously fed with HFD, (n = 5).

An increase in macrophage infiltration in WAT is a unique feature seen in HFD-induced obesity^12^,^13^. Results from a quantitative PCR (qPCR) study show that an alternating diet resulted in dramatically lower mRNA levels of macrophage markers (*F4/80, Cd68, Cd11b and Cd11c)* and inflammatory factor *(Mcp1* and *Tnfα)* genes in EWAT, compared to mice fed an HFD continuously (Fig. 4a–f). It was noted that animals on a (5 + 1) schedule also exhibited a lower level of expression of macrophage marker genes, suggesting the importance of diet alternation in blocking macrophage infiltration to WAT.

### Alternating diet improves HFD-induced hyperinsulinemia and glucose intolerance

**Figure 5:**
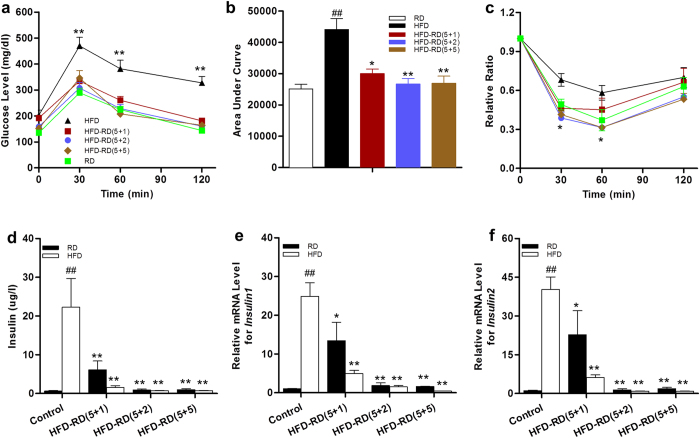
An alternating diet improved glucose tolerance and insulin sensitivity. Animals were fasted 6 h for glucose tolerance tests, or 4 h for insulin sensitivity tests. (**a**) Time-dependent blood concentration of glucose upon *IP* injection of glucose (1.5 g/kg). (**b**) Area under the curve from glucose tolerance test. (**c**) Time-dependent ratio of glucose concentration upon *IP* injection of insulin (0.75 U/kg). (**d**) Insulin level at end of 8-week feeding. (**e,f**) mRNA levels of *insulin 1* and *insulin 2* in pancreas at the end of the experiments (n = 5). ^##^*P* < 0.01 compared to mice continuously fed a regular diet, *P < 0.05, **P < 0.01 compared to mice fed continuously an HFD, (n = 10).

Animals on a continuous HFD feeding develop insulin resistance, a sign of an obesity-associated metabolic disorder^14^. To examine the effect of an alternating diet on obesity-associated insulin resistance, standard glucose tolerance tests were performed on animals at the end of the experiments. The results in Fig. 5a,b show that all animals on an alternating diet, including animals with a (5 + 1) schedule, were more sensitive to the glucose challenge compared to HFD-fed obese mice. Protection against insulin resistance by an alternating diet was also confirmed using an insulin tolerance test (ITT) (Fig. 5c). In concordance with improved insulin sensitivity, insulin levels in mice fed an alternating diet were markedly lower. Furthermore, mRNA levels of *Insulin1* and *Insulin2* in the pancreas were also lower in mice fed an alternating diet (Fig. 5d–f). Taken together, these results demonstrate that an alternating diet protects animals against HFD-induced insulin resistance and glucose intolerance.

### Alternating diet suppressed HFD-induced fatty liver

**Figure 6:**
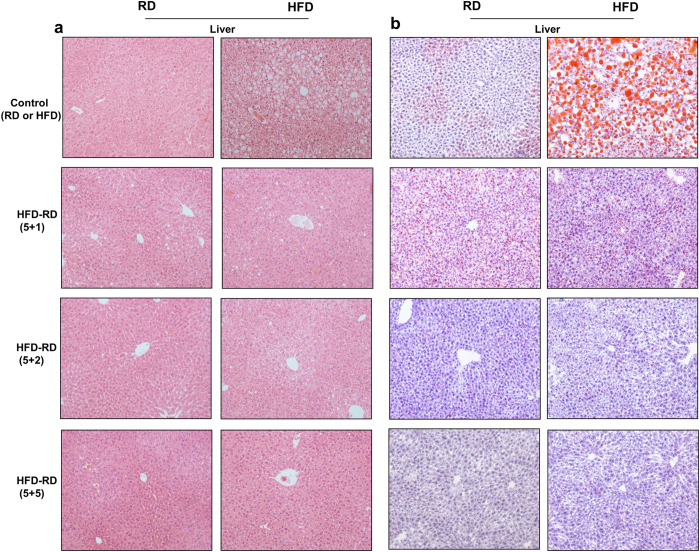
An alternating diet improved diet-induced hepatic steatosis. Mice were fed with the same or an alternating diet (5 + 1, 5 + 2, or 5 + 5) for 8 weeks and sacrificed while on either regular diet or HFD, and liver samples were collected. (**a**) Images of liver sections stained by H&E (left 2 panels). (**b**) Images of cryo-sections of the liver stained with Oil Red O (right 2 panels), 100×.

Continuous HFD feeding dramatically induced hepatic steatosis, which was evidenced by H&E staining of liver sections and confirmed by Oil red O staining (Fig. 6a,b). An alternating diet, even with a (5 + 1) schedule, markedly alleviated fatty liver compared to those animals on a continuous HFD. Animals on the (5 + 2) or (5 + 5) diet alternation showed the least vacuolation and a lower number of lipid droplets in the liver, at the level similar to that of mice fed a continuous regular diet.

**Figure 7:**
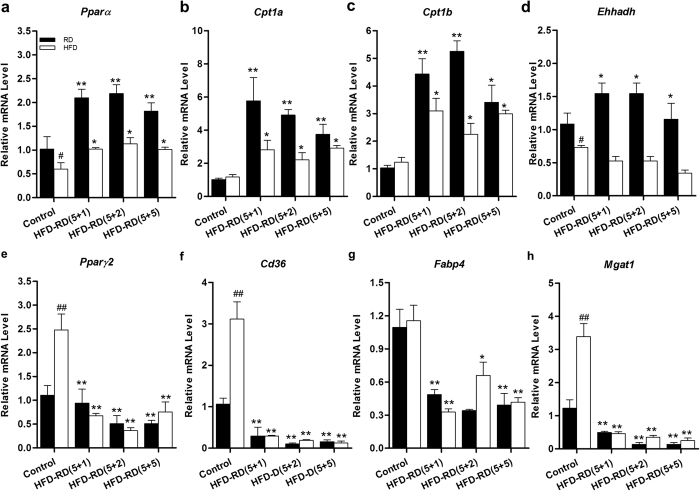
Effects of an alternating diet on expression of genes involved in hepatic fatty acid oxidation and lipid accumulation. Mice were sacrificed at the end of the experiment and total RNA was extracted from liver tissues. Real time PCR was performed to determine the mRNA levels of *Pparα* (**a**), *Cpt1a* (**b**), *Cpt1b* (**c**), *Ehhadh* (**d**), *Pparγ2* (**e)**, *Cd36* (**f**) *Fabp4* (**g**) and *Mgat1* (**h**) (^#^*P* < 0.05, ^##^P < 0.01 compared to mice continuously fed a regular diet; *P < 0.05, **P < 0.01 compared to mice continuously fed an HFD, n = 5). Abbreviations: genes for peroxisome proliferator-activated receptors (*Ppar*), carnitine palmitoyltransferase 1(*Cpt1*), enoyl-CoA, hydratase/3-hydroxyacyl CoA dehydrogenase (*Ehhadh*), cluster of differentiation 36 (*Cd36*), fatty acid binding protein 4 (*Fabp4*), and monoacylglycerol O-acyltransferase 1 (*Mgat1*).

To explore how an alternating diet repressed fat accumulation in the liver, qPCR was employed to examine the mRNA levels of the *Ppar* and its target genes since PPAR is known for its involvement in regulating hepatic lipid levels^15^. The results in Fig. 7a show that continuous HFD feeding inhibited expression of the hepatic *Pparα* gene, while animals fed an alternating diet, but sacrificed when on a regular diet in the feeding cycle, showed an increase in its mRNA level by two-fold. This was the same among animals on either the (5 + 1), (5 + 2), or (5 + 5) diet schedule. Consequently, an alternating diet, when on a regular diet cycle, evidently enhanced the expression of *Pparα* target genes, including carnitine palmitoyltransferase *Cpt1a* and *Cpt1b* expression, and key enzymes involved in fat oxidation (Fig. 7b,c). In addition, an alternating diet, when animals were on a regular diet in the feeding cycle, also increased expression of the genes enoyl-CoA and hydratase/3-hydroxyacyl CoA dehydrogenase (*Ehhadh)* (Fig. 7d).

Further studies were performed to evaluate the effect of an alternating diet on the expression of hepatic *Pparγ* and its target genes, essential for lipid accumulation. The results in Fig. 7e–h show that continuous HFD feeding markedly enhanced hepatic *Pparγ2* (2.5-fold) and its target genes such as *Cd36* (3.0-fold) and monoacylglycerol O-acyltransferase 1 (*Mgat1*, 3.4-fold). In contrast, an alternating diet with a (5 + 1), (5 + 2), and (5 + 5) feeding schedule dramatically decreased mRNA levels of the same set of genes, as observed in animals sacrificed when on either the regular or an HFD diet. The mRNA levels of these genes were lower than those of animals fed a continuous regular diet. These results suggest that an alternating diet inhibited *Pparγ*-mediated hepatic lipogenesis.

**Figure 8:**
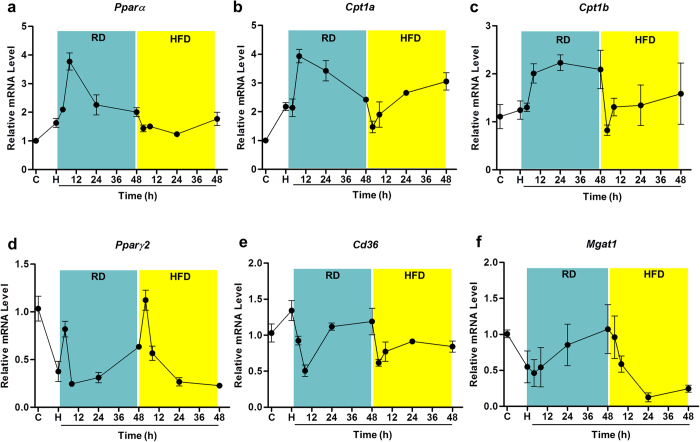
Effects of a short-term alternating diet on expression of genes involved in hepatic lipid metabolism. Mice fed a regular diet were switched to an HFD for 5 days, and then switched back to a regular diet for 2 days followed by HFD feeding for 2 additional days. After the diet switch, three mice were sacrificed at 4, 8, 24, or 48 h. Liver samples were harvested, total RNA was extracted from harvested liver samples, and qPCR was performed. Relative mRNA levels at different times after diet switch for *Pparα* (**a**), *Cpt1a* (**b**), *Cpt1b* (**c**), *Pparγ2* (**d**), *Cd36* (**e**) and *Mgat1* (**f**). (**g**) mice fed a regular diet; (**h**) mice fed an HFD; period of **RD:** animals sacrificed at 4, 8, 24, or 48 h after diet was switched from HFD to a regular diet; period of **HFD:** animals were sacrificed 4, 8, 24, or 48 h after the diet was switched from regular diet to HFD.

The results show that two days on a regular diet in each feeding cycle are sufficient to prevent HFD-induced weight gain, and significantly affect *Ppar* gene expression (Fig. 7a,e). To determine whether the changes in the *Ppar* mRNA level and its target genes were directly caused by an alternating diet, and not as a consequence of long-term and repeated cycles of diet switch, we exposed mice to an HFD for five days then switched to a regular diet for two days, or followed by two additional days on an HFD, before they were sacrificed. Data in Fig. 8 show that mRNA levels of *Pparα* and *Cpt1* were significantly higher after switching from an HFD to a regular diet. When mice were switched back to an HFD, mRNA levels of these genes were reduced but remained at a level higher than that of the control animals. Expression of *Pparγ* and its target genes, *Cd36* and *Mgat1*, were down-regulated during the process. All of these data suggest that an alternating diet is capable of enhancing gene expression involved in lipid oxidation and suppressing *Pparγ* gene expression.

### Alternating diet stabilizes and reverses preexisting obesity

**Figure 9:**
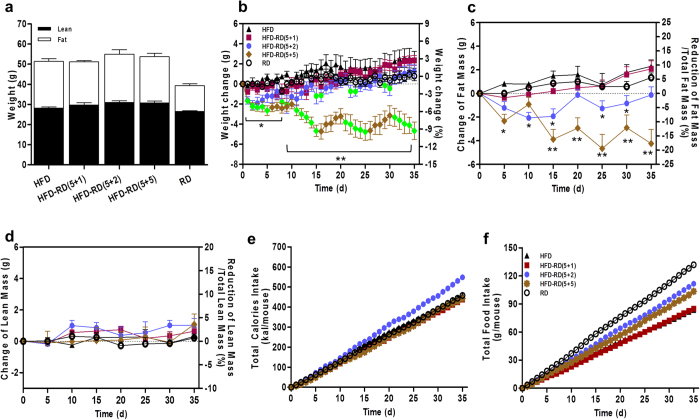
Alternating diet induced weight loss in obese mice. Age-matched obese or normal C57BL/6 mice (male, average 55 g for obese mice) were fed an HFD, regular chow, or an alternating diet for 5 weeks. Schedule for alternating diet is 5 days of HFD followed by 1d, 2d, or 5d of regular diet. (**a**) Fat and lean mass before experiments were analyzed using EchoMRI-100^TM^ System. (**b**) Weight change in continuous chow- or HFD-feeding and alternating diet-feeding groups. Green dots represent body weight when animals were on regular diet. (**c**) Fat mass and (**d**) lean mass. (**e**) Total food intake. (**f**) Total calories intake per mouse was calculated based on daily food and caloric intake (HFD: 5.49 kcal/g; regular chow: 3.46 kcal/g). Data represent mean ± SEM (n = 10). *P < 0.05, **P < 0.01 compared to mice with continuous HFD feeding.

Considering the preventive effects of an alternating diet on HFD-induced obesity, we next evaluate whether this strategy is able to induce weight loss in preexisting obesity. As shown in Fig. 9a, C57BL/6 mice become obese after 25 weeks of HFD feeding and over 40% of body composition is fat mass. We exposed these obese mice to an alternating diet (HFD for five days followed by a regular diet for one, two, or five days, then the cycle repeated) for a total of five weeks. The control animals were fed either an HFD or a regular diet continuously without a diet switch. The results in Fig. 9b show that mice on a continuous HFD gained two extra grams of body weight after five weeks. In contrast, mice with a (5 + 5) schedule progressively induced weight loss and reduced body weight by five grams, accounting for a 12% body weight reduction compared to that of control animals. The reduction of body weight came from loss of fat mass, not lean mass (Fig. 9c,d). Again, animals with a (5 + 1) schedule have a similar trend of weight gain to the control mice fed an HFD continuously. Animals with a (5 + 2) schedule induced weight loss at the beginning and returned to the original body weight later. Analysis of food consumption showed that the patterns of energy intake in obese mice are similar to the ones in the preventive study and the fluctuated energy intake was consistent in every cycle of dietary alternation (Supplemental Fig. S1). However, the total energy intake in all groups of animals is similar, with the exception of the animals with a (5 + 2) schedule, in which there was a slight increase (Fig. 9e,f).

### Alternating diet remodeled adipocytes in obese mice and reduced obesity-related inflammation

**Figure 10:**
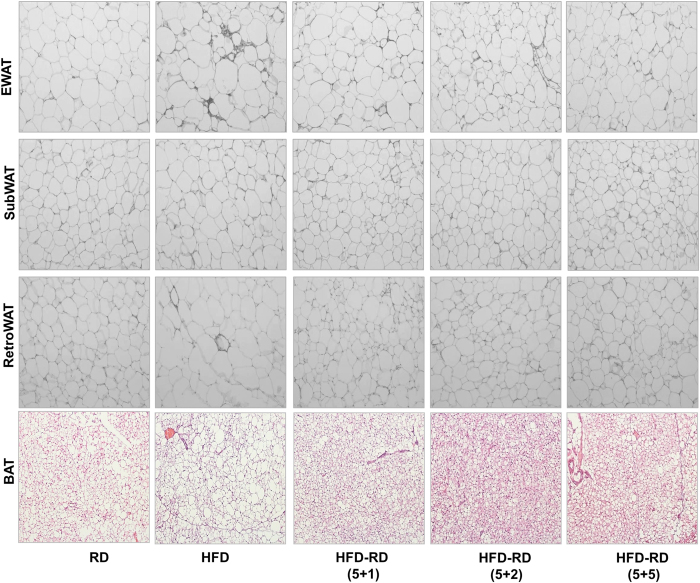
Alternating diet remodeled adipocytes in obese mice. Obese or normal male C57BL/6 mice were fed an HFD, regular chow, or an alternating diet for 5 weeks. At the end of the 5-week alternating diet feeding, epididymal white adipose tissue (EWAT), subcutaneous WAT (SubWAT), retroperitoneal WAT (RetroWAT) and BAT were collected and H&E staining was performed to examine structure of adipocytes (100×).

Consistent with the reduction in whole-body fat content under an alternating diet with a (5 + 5) schedule, H&E-stained sections of WAT, including EWAT, SubWAT, and RetroWAT, revealed smaller adipocytes compared to obese animals with a continuous HFD feeding. Moreover, large fat droplets were seen in the BAT of the control obese mice. These droplets were virtually absent in mice with diet alternation (Fig. 10). In addition, much less fat accumulation was seen in WAT and BAT, suggesting the beneficial effect of an alternating diet on adipose remodeling.

**Figure 11:**
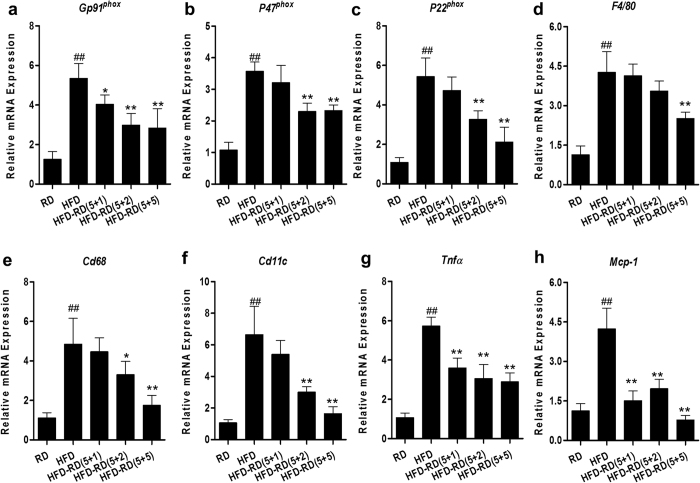
Alternating diet reduced diet-induced oxidative stress and inflammation in eWAT of obese mice. Obese or normal male C57BL/6 mice were fed an HFD, regular chow, or an alternating diet for 5 weeks. At the end of the experiments, total RNA was extracted from eWAT. Real time PCR was performed to determine the mRNA levels of genes involved in oxidative stress including (**a**) Gp91^phox^, (**b**) P47^phox^, (**c**) P22^phox^; macrophage markers (**d**) *F4/80*, (**e**) *Cd68*, (**f**) *Cd11c*, and inflammatory factors (**g**) *Tnfα* and (**h**) *Mcp1*. *P < 0.05, **P < 0.01 compared to mice with continuous HFD feeding, (n = 5).

Gene expression analysis showed that a continuous HFD feeding greatly increased oxidative stress and chronic inflammation in the obese mice, whereas an alternating diet markedly reduced these changes. This was evidenced by reduced mRNA levels of NAPDH oxidase subunit genes *gp91*^*phox*^*, p22*^*phox*^, and *p40*^*phox*^, macrophage marker genes (*F4/80, Cd68, Cd11b* and *Cd11c*), and genes encoding inflammatory factors (*Mcp1* and *Tnfα*). The greatest change was found in animals with a (5 + 5) alternating diet schedule (Fig. 11).

### Alternating diet improved hepatic steatosis and insulin sensitivity in obese mice

**Figure 12:**
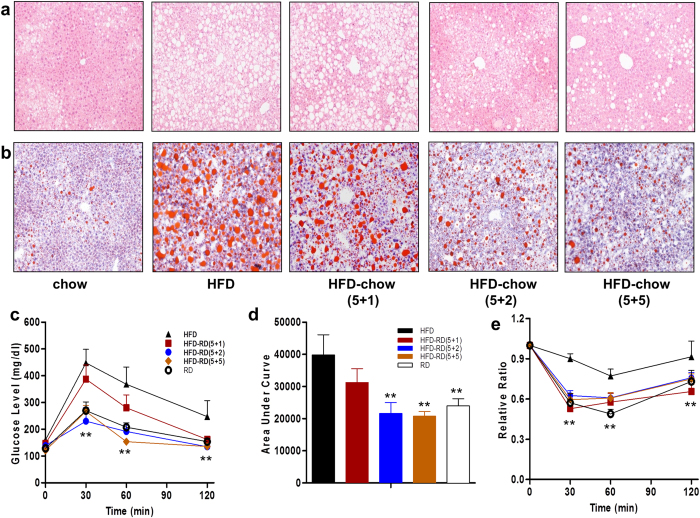
Alternating diet improved hepatic steatosis and insulin sensitivity in obese mice. Liver structures were determined by H&E (**a**), and the levels of lipid accumulation were detected by Oil Red O staining (**b**) (100×). (**c**) Time-dependent blood concentration of glucose upon IP injection of glucose (1.5 g/kg).(**d**) Area under the curve from glucose tolerance test. (**e**) Time-dependent ratio of glucose concentration upon IP injection of insulin (0.75 U/kg). *P < 0.05, **P < 0.01 compared to mice with continuous HFD feeding, (n = 10).

Obesity is often accompanied by lipid overload in the liver. However, H&E staining of liver sections revealed that an alternating diet significantly lowered the lipid level in the liver compared to the mice from a continuous HFD feeding. Oil Red O staining also confirmed these results (Fig. 12a,b). Results from real-time PCR analysis revealed that an alternating diet significantly enhanced PPARα and its target gene expression, which was lower in obese mice (Supplemental Fig. S2). Because an alternating diet reduced fat accumulation and protected from adipose inflammation, glucose tolerance and insulin sensitivity tests revealed that it improved insulin sensitivity in obese mice as well (Fig. 12c–e). Overall, these results demonstrate that an alternating diet improves obesity-related hepatic steatosis and insulin sensitivity.

## Discussion

In the present study, we have evaluated a novel procedure of alternating diet to control HFD-induced obesity in mice. We demonstrate that an alternating diet with a (5 + 2) and (5 + 5) schedule successfully blocked weight gain and fat mass expansion (Fig. 1a–e), without significantly affecting total energy intake (Fig. 2e). The alternating diet dramatically suppressed macrophage infiltration into adipose tissue (Fig. 4), blocked HFD-induced insulin resistance (Fig. 5), and inhibited development of fatty liver (Fig. 6). The pleiotropic beneficial effects were associated with lowering energy conversion efficiency, stimulating expression of genes involved in hepatic fat oxidation, and suppressing expression of genes responsible for lipid storage (Figs 7 and 8). Moreover, an alternating diet with a (5 + 5) schedule induced weight loss in obese mice without affecting total energy intake (Fig. 9b,e) and improved obesity-associated metabolic abnormalities, including adipose inflammation, fatty liver, and insulin resistance (Figs 11 and 12).

Dietary manipulation is the basal and first-line intervention in combating obesity and obesity-associated metabolic disorders, due to its ease of access and lower cost. Thus, previous studies focused on limiting caloric intake^6^,^7^,^8^ or feeding time^16^,^17^, whereas this current study stresses the importance of providing animals with free access to a diet composed of either high or normal fat content. Our results show that alternating an HFD and a regular diet in animals is effective in controlling weight gain and metabolic complications. The benefits of an alternating diet are primarily correlated to diet-based fluctuation in energy intake and lower energy conversion efficiency. As shown in Fig. 2b–d and Fig. S1, daily calorie intake dropped dramatically when switching from an HFD to a regular diet. A significant increase in energy intake was seen when switching back to an HFD and the patterns of energy intake were consistent for the entire feeding period. The fluctuating energy intake between a regular diet and an HFD may physiologically mimic the food intake of some terrestrial mammals with seasonal changes in food intake and body weight^18^. This dynamically affects expression of genes under the control of PPAR responsible for regulation of fat oxidation and storage.

The alternating diet dramatically inhibited diet-induced hepatic lipid accumulation, as evidenced by H&E and Oil Red O staining of liver sections (Figs 6 and 12a,b). This beneficial effect is attributed to the enhancement of PPARα-induced lipid oxidation and suppression of PPARγ-mediated fat storage, irrespective of long-term or short term dietary alternation. Fluctuation of energy intake evidently stimulates hepatic lipid oxidation, which was demonstrated by enhanced *Pparα* gene expression and its target genes *Cpt1a, Cpt1b*, and *Ehhadh*, coding for essential enzymes for fatty acid oxidation (Fig. 7a–d). PPARα is a master regulator in lipid metabolism and is expressed in the tissues that are active in fatty acid oxidation, including the liver, BAT, heart, and kidneys^19^. PPARα-null mice showed inhibition of fatty acid oxidation and displayed severe hepatic steatosis when animals were challenged with an HFD or fasting^20^. Conversely, activation of PPARα by an agonist ameliorated hepatic lipid accumulation induced by alcohol^21^ or methionine and choline-deficient diet^22^,^23^, suggesting that PPARα plays a protective role in hepatic steatosis. Our results show that elevated expression of *Pparα* and its target genes is more evident in animals on a regular diet during an alternating diet feeding, which may be due to the temporary fasting that happened in this stage. As shown in Fig. 2, mice took in much less energy when switching from an HFD to a regular diet. We labeled this a “self-fasting” period. It is known that fasting is a strong activator of the PPARα-dependent signaling pathway^20^. Therefore, dietary alternation dynamically activates PPARα-dependent signaling and promotes the utilization of hepatic lipids, protecting the mice from developing fatty liver.

PPARγ is a transcription factor regulating genes responsible for lipid storage and adipocyte differentiation^24^. The overexpression of *Pparγ* exacerbated liver fat accumulation^25^, whereas disruption of hepatic *Pparγ* protected mice against hepatic steatosis^26^,^27^,^28^. Consistent with previous studies^29^,^30^, our results show that an HFD significantly elevated liver *Pparγ2* gene expression. However, the alternating diet dramatically inhibited *Pparγ* expression at the transcript level, even with one day on the regular diet and five days on the HFD. Accordingly, the alternating diet significantly suppressed expression of PPARγ target genes, including *Cd36*, *Fabp4*, and *Magt1*, whose mRNA levels were even lower than those of regular diet-fed mice. *Cd36* codes for a transporter that is responsible for moving free fatty acids into the liver, while *Mgat1* is involved in incorporating fatty acids into triglycerides^31^,^32^. Previous studies have shown that the elevated mRNA levels of these genes are directly linked to hepatic steatosis^33^,^34^. Therefore, by targeting the hepatic PPARγ pathway, the alternating diet also exerts its protective function against obesity-induced hepatic steatosis.

Long-term exposure to excessive nutrients places a heavy burden on adaptive responses that lead to continuous chronic inflammation and oxidative stress, resulting ultimately in a metabolic imbalance^35^,^36^. Previous studies have shown that HFD-induced obesity is associated with infiltration of inflammatory cells in WAT, as evidenced by the presence of crown-like structures^12^,^13^. However, the crown-like structures are barely visible in mice fed an alternating diet. In addition, results from qPCR analysis also show that an alternating diet with a (5 + 2) and a (5 + 5) schedule resulted in lower mRNA levels of macrophage marker genes and inflammatory factors in EWAT. These results prove that an alternating diet significantly inhibited macrophage infiltration and chronic inflammation. Mechanistically, continuous HFD feeding induces adipocyte hypertrophy that enhances metabolic stresses and the rate of adipocyte death, accordingly triggering the macrophage infiltration and inflammation^37^,^38^. An alternating diet may dynamically reduce adipocyte lipid storage, relieve cell stress, and attenuate the pro-inflammatory response, thus maintaining metabolic homeostasis.

In addition, an alternating diet had far-reaching effects on glucose metabolism and insulin sensitivity. Mice with a continuous HFD feeding showed significant hyperglycemia and hyperinsulinemia, while an alternating diet suppressed insulin levels and pancreatic insulin gene expression. Irrespective of adiposity, when mice fed an alternating diet were challenged with glucose, they were able to restore normoglycemia much faster than mice fed a continuous HFD feeding and were more sensitive to the insulin stimulation, suggesting that an alternating diet is capable of maintaining glucose homeostasis. Insulin resistance in a continuous HFD feeding is closely related to long-term nutrient excess, microhypoxia, and chronic inflammation in adipose tissue, specifically with recruitment and activation of macrophages^39^. Growing evidence demonstrates that chronic low-grade tissue inflammation is an important contributor to the development of obesity-induced insulin resistance. The lipid overload in adipose tissue and in the liver increases stress response, resulting in the recumbence of immune cells and the stimulation of inflammatory pathways. Subsequently, insulin influence is reduced in the stressed tissues through inhibiting insulin-signaling transduction, causing cellular insulin resistance^11^,^40^. Insulin resistance also induces the expression of key inflammatory cytokine genes such as interleukin 1-beta in macrophages due to the interaction of NF-κB and FoxO1, a key transcription factor mediating insulin action^41^. Dietary alternation efficiently regulates lipid metabolism and limits inflammation in adipose tissue, exerting beneficial effects on insulin sensitivity.

In summary, the results presented in this study clearly show that an alternating diet is an effective strategy in controlling HFD-induced obesity, hepatic lipid accumulation, glucose intolerance, and inflammation. It de-emphasizes controlling caloric intake, thus making it an attractive and easily adoptable lifestyle modification. Future studies should explore the possible metabolic regulators and illustrate the underlying mechanisms. Meanwhile, it is worth investigating whether the physiological observations found in animals apply to humans.

## Materials and Methods

### Materials

High-fat diet (F3282, 60% kJ/fat, 26% kJ/carbohydrate, 14% kJ/protein) was purchased from Bio-serv (Frenchtown, NJ). The regular chow (Prolab® Isopro® RMH 3000, 14% kJ/fat, 60% kJ/carbohydrate, 26% kJ/protein) was from LabDiet (St Louis, MO). TRIZOL reagent and SuperScript® III First-Strand Synthesis System are from Life Technologies (Grand Island, NY). RNeasy Lipid Tissue Kit was from QIAGEN (Valencia, CA). SYBR® Green FastMix was acquired from Quanta BioSciences (Gaithersburg, MD). Oil Red O solution was obtained from Electron Microscopy Science (Hatfield, PA). Mercodia Insulin ELISA kit was from Mercodia Developing Diagnostics (Winston Salem, NC). TRUEtrack glucometer and test strips were purchased from Nipro Diagnostics (Fort Lauderdale, FL).

### Animals and treatment

All procedures performed on animals were approved by the Institutional Animal Care and Use Committee at the University of Georgia and the methods were carried out in accordance with the approved protocol #A2014-07-008-Y1-A0. C57BL/6 mice (male) were purchased from Charles River (Wilmington, MA). For the prevention study, two groups of C57BL/6 mice with a body weight of 25 g were fed an HFD or regular chow continuously for eight weeks, while three groups of mice on an alternating diet (10 per group) were first fed five days of an HFD followed by either one, two, or five days of a regular diet. They were then switched back to five days of HFD feeding to start the second cycle. The repeating feeding cycles were maintained for a total of eight weeks. On day 56, five mice were sacrificed, and the remaining five were placed on the opposite diet to that which they were on at day 56 for one day and then sacrificed. For the treatment study, age-matched obese or normal C57BL/6 mice (male, average 55 g for obese mice) were fed an HFD, a regular diet, or an alternating diet for five weeks and sacrificed on day 36. Body weight and food intake were monitored daily and their body composition was determined using EchoMRI-100^TM^ from Echo Medical Systems (Houston, TX).

### Histochemical analysis

After the mice were sacrificed, the liver, epididymal white adipose (EWAT), and brown adipose tissues (BAT) were collected and fixed in 10% neutrally buffered formalin, which were then embedded in paraffin and sectioned (6 μm). Hematoxylin and eosin (H&E) staining was performed using a commercial kit (BBC Biochemical, Atlanta, GA) following the manufacturer’s protocol. For Oil Red O staining, liver samples were freshly collected and immediately frozen in liquid nitrogen. Frozen sections were made at 8 μm in thickness using a Cryostat, stained with 0.2% Oil Red O in 60% of isopropanol for 15 min, counterstained with haematoxylin, and then washed three times. Microscopic examination was conducted and photographs were taken under a regular light microscope.

### Determination of insulin level and glucose homeostasis

Blood samples were collected 4 h after fasting. Insulin levels in plasma were measured using commercial assay kits according to the manufacturer’s instructions. A glucose tolerance test and an insulin tolerance test were performed between 50–56 days (prevention study) or 25–30 days (treatment study). For the glucose tolerance test, mice were injected intraperitoneally with glucose at 1.5 g/kg body weight after fasting for 6 h. Blood samples were taken at varying time points and glucose concentrations were measured using a glucometer. For the insulin tolerance test, mice were fasted for 4 h and then administrated insulin intraperitoneally (0.75 U/kg, Eli Lilly, Indianapolis, IN), after which blood glucose concentrations were determined.

### Gene expression analysis by real-time PCR

Total RNA was isolated from the mouse EWAT, liver, and pancreas using an RNeasy kit or the TRIZOL reagent. Real-time PCR was carried out using SYBR Green as an indicator using the ABI StepOne Plus Real-Time PCR system. Briefly, 2 μg of total RNA were used for the first strand cDNA synthesis, as recommended by the manufacturer. PCR was performed for 40 cycles at 95 °C for 15 sec and 60 °C for 1 min. The data were normalized using GAPDH mRNA as an internal control. The primer sequences employed are summarized in Table S1.

### Statistical analysis

Data were analyzed using GraphPad Prism 6 (GraphPad Software, San Diego, CA). Statistical analysis was performed by two-way ANOVA followed by a Duncan’s multiple range post hoc tests. Data are reported as mean ± SEM with statistical significance set at *P* < 0.05.

## Additional Information

**How to cite this article**: Ma, Y. *et al.* Alternating Diet as a Preventive and Therapeutic Intervention for High Fat Diet-induced Metabolic Disorder. *Sci. Rep.* **6**, 26325; doi: 10.1038/srep26325 (2016).

## Supplementary Material

Supplementary Information
