# Supplementary material for: Alternating Diet as a Preventive and Therapeutic Intervention for High Fat Diet-induced Metabolic Disorder
Source: Sci Rep. 2016 May 18;6:26325. doi: 10.1038/srep26325 (PMC4870701; doi:10.1038/srep26325)
Supplement: Supplementary Information [file srep26325-s1.pdf]

Alternating Diet as a Preventive and Therapeutic Intervention for High Fat Diet-induced Metabolic Disorder

Yongjie Ma[1], Mingming Gao[1] and Dexi Liu[1]*

Supplementary Table S1

Supplementary Figure S1 and Figure Legend

Supplementary Figure S2 and Figure Legend

Supplemental Data

Table S1. Primer sets for real time PCR analysis of gene expression

Name	**Forward sequence**	**Reverse sequence**
Pparγ1	GGAAGACCACTCGCATTCCTT	GTAATCAGCAACCATTGGGTCA
Pparγ2	TCGCTGATGCACTGCCTATG	GAGAGGTCCACAGAGCTGATT
Cd36	CCTTAAAGGAATCCCCGTGT	TGCATTTGCCAATGTCTAGC
Fabp4	AAGGTGAAGAGCATCATAACCC	TCACGCCTTTCATAACACATTCC
Mgat1	TGGTGCCAGTTTGGTTCCAG	TGCTCTGAGGTCGGGTTCA
Pparα	TGTCGAATATGTGGGGACAA	AATCTTGCAGCTCCGATCAC
Acox1	CCGCAACCTTCAATCCAGAG	CAAGTTCTCGATTTCTCGACGG
Cpt1a	CTCCGCCTGAGCCATGAAG	CACCAGTGATGATGCCATTCT
Cpt1b	GGTCTCTTCTTCAAGGTCTG	CGAGGATTCTCTGGAACTGC
Acadl	TCTTTTCCTCGGAGCATGACA	CAGACCTCTCTACTCACTTCTCCAG
Acadm	TTGAGTTGACGGAACAGCAG	GCCCCAAAGAATTTGCTTCAA
Ehhadh	ATGGCTGAGTATCTGAGGCTG	ACCGTATGGTCCAAACTAGCTT
Fgf21	CTGCTGGGGGTCTACCAAG	CTGCGCCTACCACTGTTCC
Insulin1	CACTTCCTACCCCTGCTGG	ACCACAAAGATGCTGTTTGACA
Insulin2	GCTTCTTCTACACACCCATGTC	AGCACTGATCTACAATGCCAC
F4/80	CCCCAGTGTCCTTACAGAGTG	GTGCCAGAGTGGATGTCT
Cd68	CCATCCTTCACGATGACACCT	GGCAGGGTTATGAGTGACAGTT
Cd11b	ATGGACGCTGATGGCAATACC	TCCCCATTCACGTCTCCCA
Cd11c	ACGTCAGTACAAGGAGATGTTGGA	ATCCTATTGCAGAATGCTTCTTTACC
Mcp1	ACTGAAGCCAGCTCTCTCTTCCTC	TTCCTTCTTGGGGTCAGCACAGAC
Gapdh	AGGTCGGTGTGAACGGATTTG	TGTAGACCATGTAGTTGAGGTCA

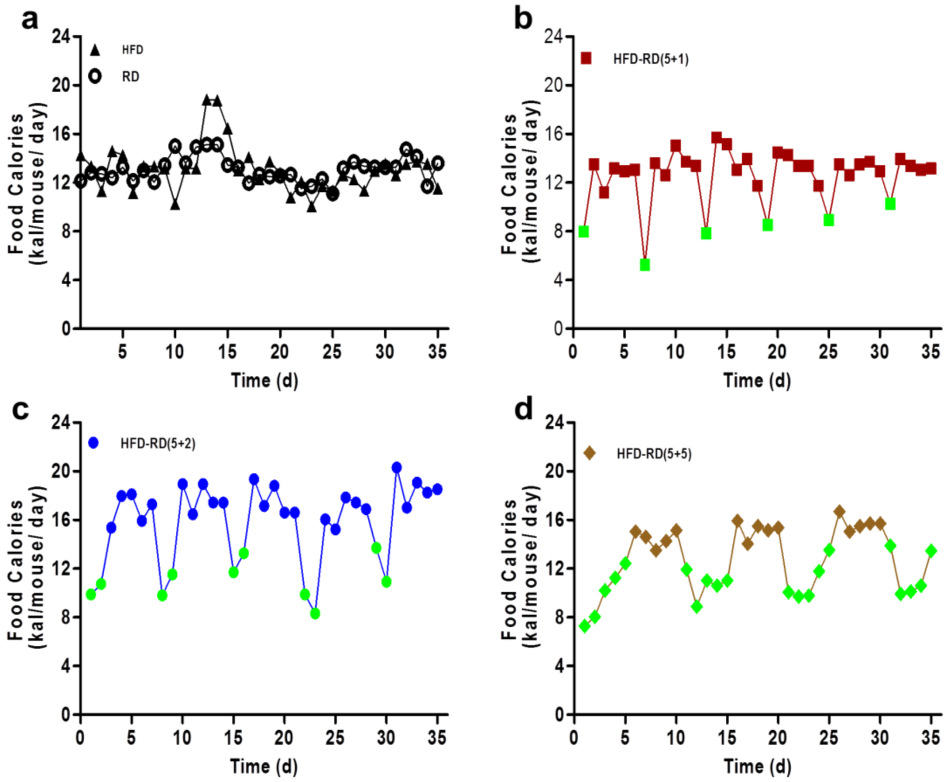

Figure S1. Effects of an alternating diet on daily calorie intake in obese mice. C57BL/6 obese mice and age-matched normal mice were fed a HFD and regular diet continuously (A), or an alternating diet with a schedule of 5+1 (B), 5+2 (C) or 5+5 (D). The green dots in panels B, C and D represent caloric intake of mice when on regular chow. Daily caloric intake was calculated based on daily food intake. HFD: 5.49 kcal/g; chow food: 3.46 kcal/g. Values were calculated from food intake of 5 animals.

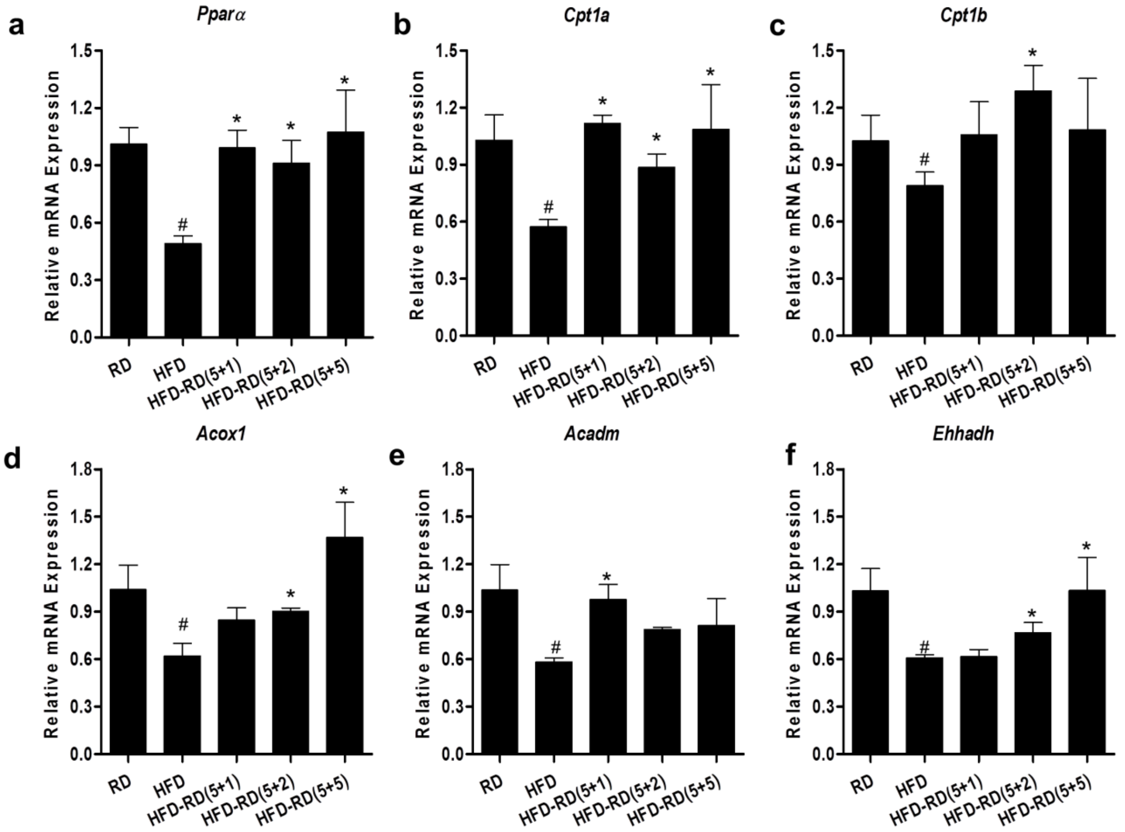

Figure S2. Alternating reversed reduction of genes expression involved in β-oxidation . Mice were sacrificed at the end of experiment and total RNA was extracted from liver tissues. Real time PCR was performed to determine the mRNA levels of *Pparα* (**A**), *Cpt1a* (**B**), *Cpt1b* (**C**), *Acox1* (**D**), *Adadm* (**E**), and *Ehhadh* (**F**). $^{\#}P<0.05$, $^{\#\#}P<0.01$ compared to mice continuously fed a regular diet; *P<0.05, **P<0.01 compared to mice continuously fed an HFD (n=5).
